# Comparative and phylogenetic analyses of the chloroplast genomes of *Filipendula* species (Rosoideae, Rosaceae)

**DOI:** 10.1038/s41598-023-45040-3

**Published:** 2023-10-18

**Authors:** Shu-Dong Zhang, Li-Zhen Ling

**Affiliations:** School of Biological Science and Technology, Liupanshui Normal University, Liupanshui, 553004 China

**Keywords:** Evolution, Plant sciences

## Abstract

Genus *Filipendula* (Rosoideae, Rosaceae) comprises about 15 species and mainly distributed in Northern Hemisphere. The phylogenetic relationships based on the nrITS marker are not consistent with the traditional taxonomic systems of the genus. Here, we first analysed the complete chloroplast (cp) genomes of seven *Filipendula* species (including two varieties of *F. palmate*). Our results indicated that the cp genomes of *Filipendula* species had few changes in size, ranging from 154,205 bp to 154,633 bp and the average of 36.63% GC content. A total of 126 annotated genes had the identical order and orientation, implying that the cp genome structure of *Filipendula* species was rather conserved. However, the cp genomes of *Filipendula* species exhibited structural differences, including gene loss, transposition and inversion when compared to those of other genera of Rosoideae. Moreover, SSRs with the different number were observed in the cp genome of each *Filipendula* species and sequence divergence mainly occurred in noncoding regions, in which four mutational hotspots were identified. In contrast, only two positive selection genes (*matK* and *rps8*) were found. Phylogenetic and molecular-dating analysis indicated that *Filipendula* species were divergent from other genera of Rosoideae at about 82.88 Ma. Additionally, *Filipendula* species from East Asia were split at about 9.64 Ma into two major clades. These results provide a basis for further studying the infrageneric classification of *Filipendula*.

## Introduction

Chloroplast (cp) is a specialized eukaryotic organelle and its genetic materials are mainly maternally inheritance, in which a core set of genes have originated from the cyanobacterial ancestor and are mostly involved in photosynthesis and metabolic processes^1–4^. Chloroplast genome has a small size and is roughly 120–180 kilobases in length^5^. The advancement of modern sequencing technologies has boosted the study of chloroplast genetics and genomics. Insights into chloroplast genome sequences have revealed considerable sequence and structural variations occurred within and between plant species. For example, three types of mutations, including gene/intron loss, inverted repeat changes and inversions in the land plant chloroplast genomes can lead to the gene order changes and are often referred to as structural changes or rearrangements^5^. To date, chloroplast genomes have been widely utilized as markers for studying the species identification, phylogenetic and population analyses^6–8^.

*Filipendula* Mill. (Rosoideae, Rosaceae) is a perennial herbaceous plant and contains approximately 15 species, which generally grows in the high mountain of the temperate regions^9^. The geographic distribution of *Filipendula* mainly covers East Asia, Europe and North America^10^. *Filipendula* species have long been utilized for medicinal purposes and most published papers have focused on the medicinal properties of these plants^11,12^. Their aerial parts (leave and flower) and underground organs (roots) are good resources of bioactive substances, including tannins, polyphenolic acid and essential oils, which have antioxidant, anticancer, anti-inflammatory, gastroprotective, anti-hyperalgesic, anti-genotoxic, and hepatoprotective effects^13,14^. Besides, the leaves of *Filipendula* can be processed into the herbal tea in Russia and other Siberia countries, which is used to relieve influenza and gout, to clean wounds and eyes^15^.

The classification of genus *Filipendula* is confused all the time^16^. Juzepczuk^16^ has divided this genus into three subgenera and two sections mainly based on the indigenous species. Afterward, Shimizu^17^ revised the former taxonomic system and classified 15 species of the whole genus into two monotypic subgenera (*Hypogyna* T. Shimizu and *Filipendula*) and one large subgenus (*Ulmaria* Moench) with four sections (*Ulmaria* Hill, *Albicoma* Juz., *Sessilia* T. Shimizu and *Schalameya* Juz.). In 1967, Sergievskaya amended the two former systems and divided the genus into four subgenera, including three subgenera of Shimizu’ system and subgenus *Aceraria* of Juzepczuk’s system^16^. Of these four subgenera, only Shimizu’s sect. *Ulmaria* was retained within subgenus *Ulmaria* and the remaining sections were transferred into subgenus *Aceraria*. In the last taxonomic revision of the genus, Schanzer^9^ divided the genus into four sections: *Hypogyna*, *Schalameya*, *Albicoma* and *Filipendula* mainly based on the morphological and geographic data. Therefore, the four systems are incongruent with each other to a certain extent and the names of some species in the different systems are still used.

To date, limited studies have been documented on *Filipendula* diversity and phylogenetic analysis. Only few studies have reported that isozymes^18^ and microsatellites^19^ can be used as markers to assess genetic variations in *F. vulgaris*. Investigations of the phylogeny of Rosoideae or Rosaceae have revealed that *Filipendula* as monophyly is sister to the rest of the subfamily Rosoideae^20–22^. Several evidence have revealed that the species in the basal lineage exhibited the unique chloroplast structure. For example, a single inversion as the powerful phylogenetic marker identified the basal members of the Asteraceae^23^. In a second case, two inversions and an expansion of the IR clarified the basal nodes in leptosporangiate ferns^24^. Whether did this phenomenon occur between *Filipendula* and other genera of the subfamily Rosoideae? However, the basic knowledge of the chloroplast genome in *Filipendula* is absent and the chloroplast phylogeny of *Filipendula* species has not been reported until now. Moreover, the infrageneric phylogenetic relationships of *Filipendula* was only analysed using one nucleotide segment (ITS)^10^. Therefore, the present study aimed to provide the unprecedented chloroplast genome data for comparative analysis, to reconstruct the infrageneric phylogeny of *Filipendula* based on eight cp genomes (*F. vestita, F. ulmaria, F. palmata* (including two varieties, *F. palmata* var. *palma* and *F. palmata* var. *glabra*), *F. angustiloba, F. vulgaris*, *F. camtschatica* and *F. multijuga*) and to explore evolutionary history of this genus.

## Results

### Characterization and structural analyses of eight *Filipendula* cp genomes

**Figure 1:**
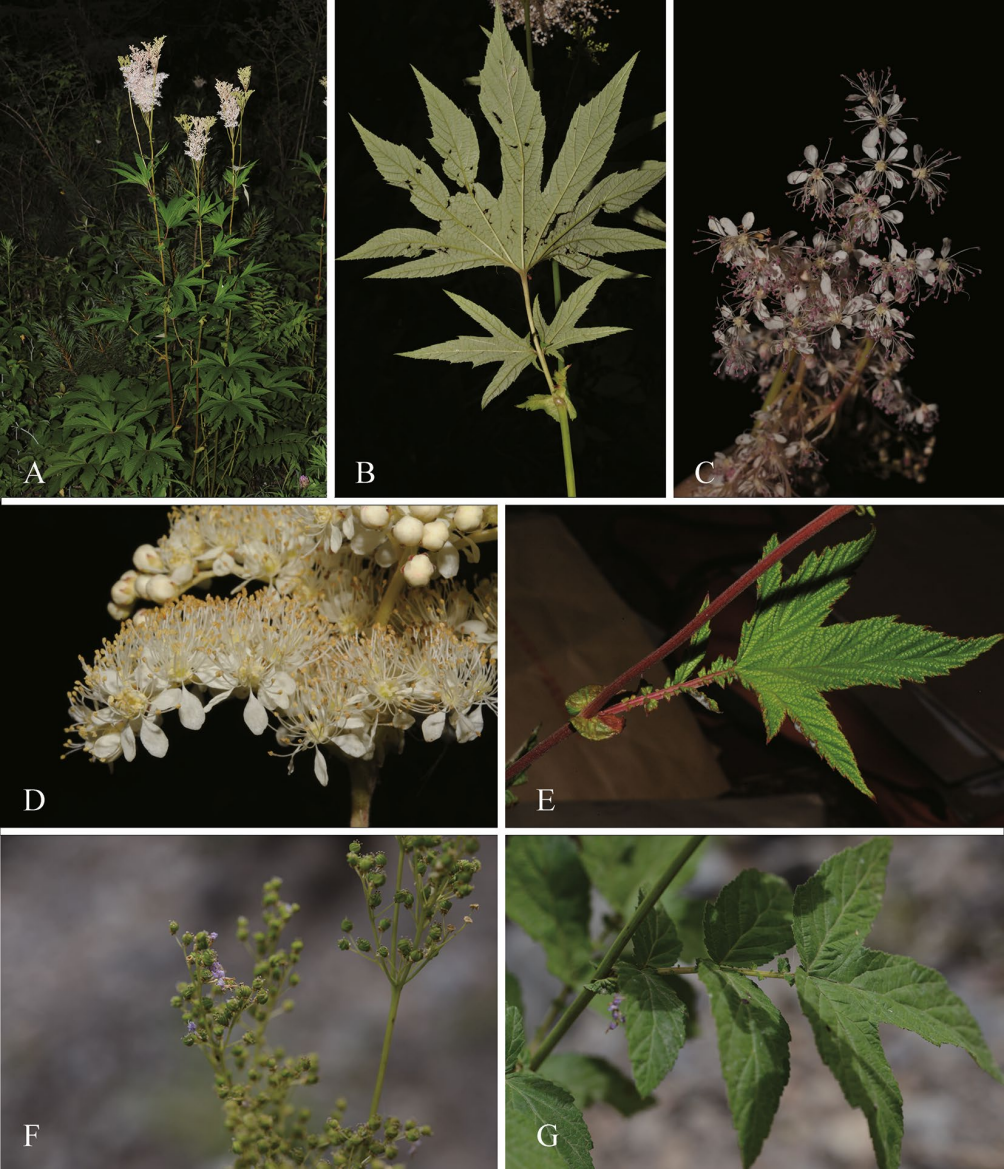
Photograph images of *Filipendula palmata* var. *palmata* (A-C), *F. vestita* (D and E) and *F.* *ulmaria* (F and G). Photos by Jie Cai, Ting Zhang and Ji-Dong Ya from Kunming Institute of Botany, Chinese Academy of Sciences.

**Figure 2:**
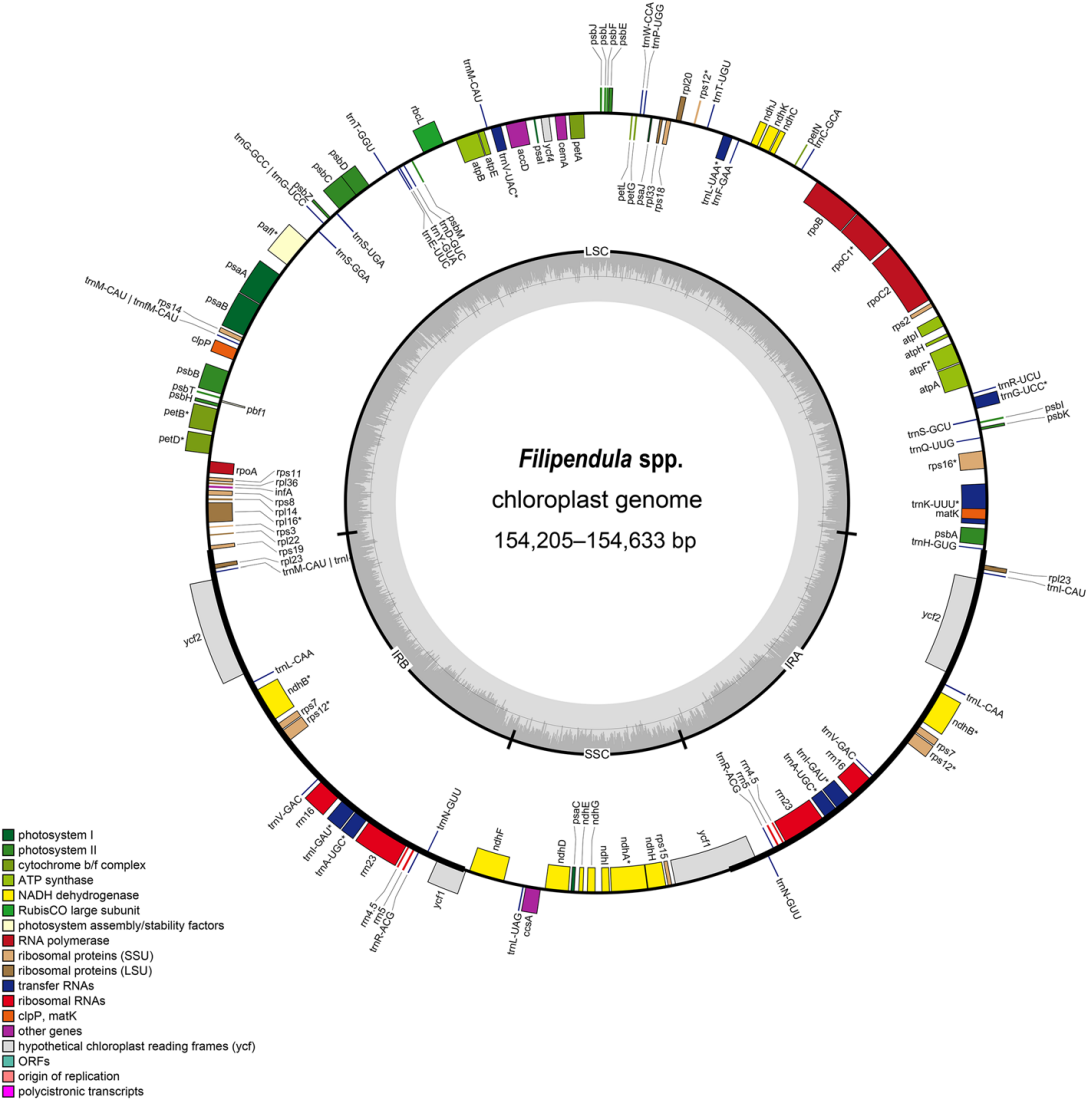
Circular map of *Filipendula* chloroplast genome. The inner grey circle indicates the GC content of each genome position. Genes in the inner circle of the genomic map are transcribed clockwise and vice versa.

In this study, eight assembled cp genomes from seven *Filipendula* species in which *F. palmata* had two varieties (Fig. 1), had an average size of 154,522 bp (ranging from 154,205 bp-154,633 bp) and 36.63% GC content (Table 1). These eight cp genomes were divided into four regions and two copies of an inverted repeat (IR) separated large and small single copy regions (LSC and SSC), respectively (Fig. 2). The four regions formed the typical circular structure and varied a little in size, in which the LSC region had a largest size, ranging from 82,851 bp to 83,295 bp, followed by the IR region (from 27,093 bp to 27,286 bp) (Fig. 2 and Table 1). In addition, a total of 126 genes were annotated in each *Filipendula* cp genome except for *F. camtschatica*, including 81 protein-coding genes (PCGs), 37 tRNA and 8 rRNA genes. It was worth noting that the gene number had reduced by one because *rpl14* was not found in *F. camtschatica* (Table 1). The majority of PCGs were involved in the photosynthesis and metabolism (Table S1). Of all genes, 16 duplicated genes were identified in the IR region, and 16 genes (*petB*, *petD*, *atpF*, *ndhA*, *ndhB*, *rpoC1*, *rps16*, *rps12*, *rpl16*, *trnA-UGC*, *trnG-UCC*, *trnI-GAU*, *trnK-UUU*, *trnL-UAA*, *trnV-UAC*, *ycf3*) had the introns, in which two genes (*ycf3 and rps12*) had two introns and the rest of them had one intron (Table S1).

**Table 1:** Summary of complete plastomes of *Filipendula* species.

Species	PCG	tRNA	rRNA	Total number	Length (bp)				GC content (%)
					Plastome	LSC	IR	SSC	
*F. angustiloba*	81	37	8	126	154,624	83,295	27,121	17,087	36.66
*F. camtschatica*	80	37	8	125	154,205	82,851	27,188	16,978	36.64
*F. multijuga*	81	37	8	126	154,633	83,173	27,286	16,888	36.69
*F. palmata* var. *glabra*	81	37	8	126	154,622	83,280	27,121	17,100	36.66
*F. palmata* var. *palmata*	81	37	8	126	154,624	83,295	27,121	17,087	36.66
*F. ulmaria*	81	37	8	126	154,464	83,154	27,093	17,124	36.63
*F. vestita*	81	37	8	126	154,524	83,218	27,101	17,104	36.53
*F. vulgaris*	81	37	8	126	154,483	83,323	26,994	17,172	36.53

PCG indicates protein-coding gene.

**Figure 3:**
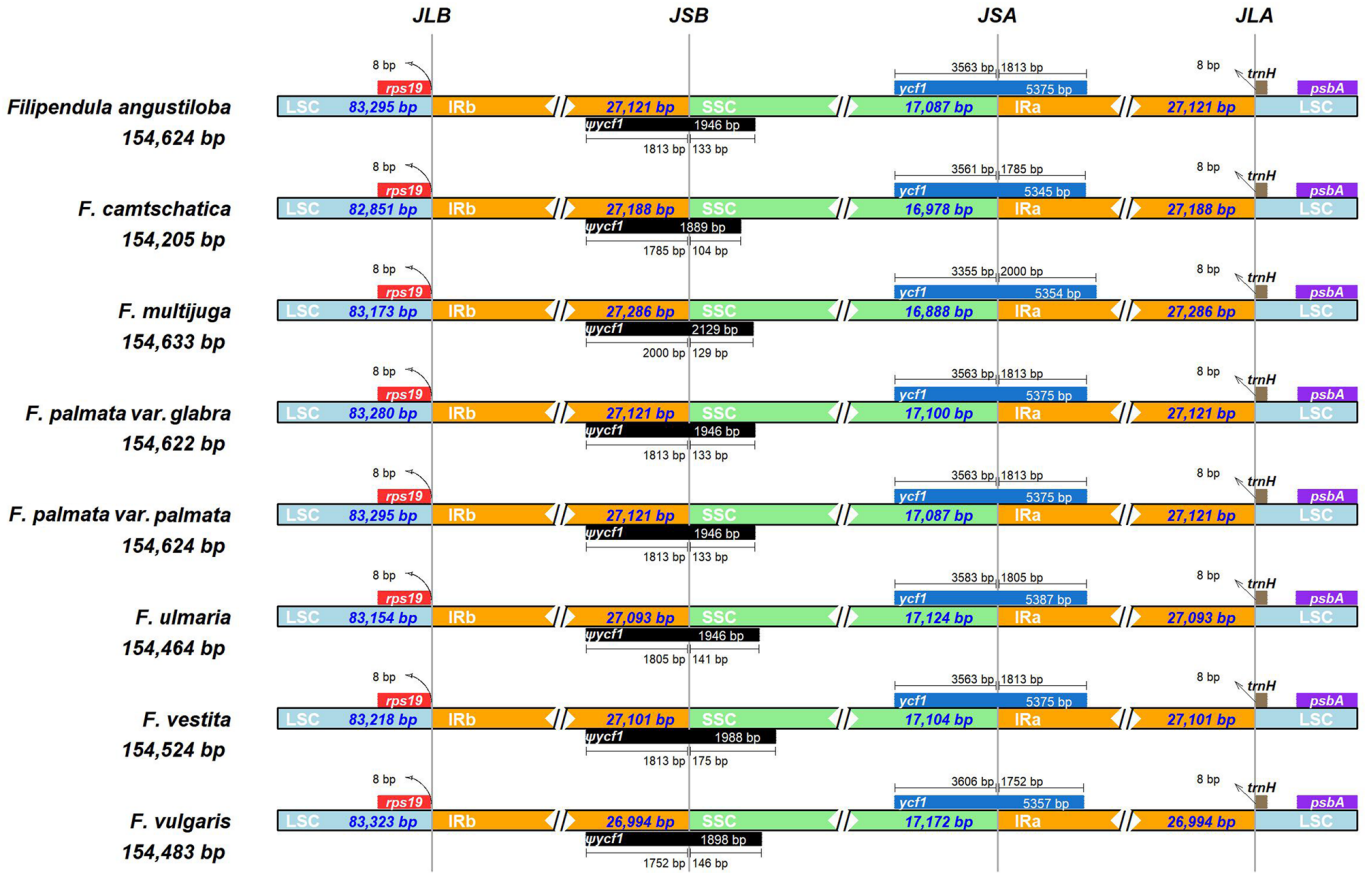
Comparison of the border regions of four chloroplast genome parts among *Filipendula* species.

To further analyse the structure of eight *Filipendula* cp genomes, multiple alignments were conducted, and the results indicated that there were an identical gene order and orientation across these tested *Filipendula* species (Figure S1), which was consistent with the result of circular map of *Filipendula* cp genome (Fig. 2). Early findings have indicated that the variations of IR play an important effect on the stability of plastome structure^5,25^. In this study, a comprehensive comparison of the IR/SC boundaries were analysed among eight *Filipendula* cp genomes. The result indicated that the boundaries of IR/LSC were very conserved, LSC/IRb/a (JLB/A) boundaries were flanked *rps19* and *trnH* with a length of 8 bp away from the 5’ end and 3’ end of these two genes, respectively (Fig. 3). In contrast, the IR/SSC junctions showed the few differences. *ψycf1* and *ycf1* separately span the boundaries of IRb/SSC (JSB) and IRa/SSC (JSA). Two flanked distances of the junction point between *ψycf1* and JSB or *ycf1* and JSA exhibited the different lengths in these two genes because the lengths of *ψycf1* and *ycf1* occurred a few changes in *Filipendula* species (Fig. 3). Therefore, the nearly unchanged IR might facilitate the stability of plastome structure of this genus. Altogether, these results demonstrated that the cp genome structure was evolutionarily conservative in *Filipendula*.

**Figure 4:**
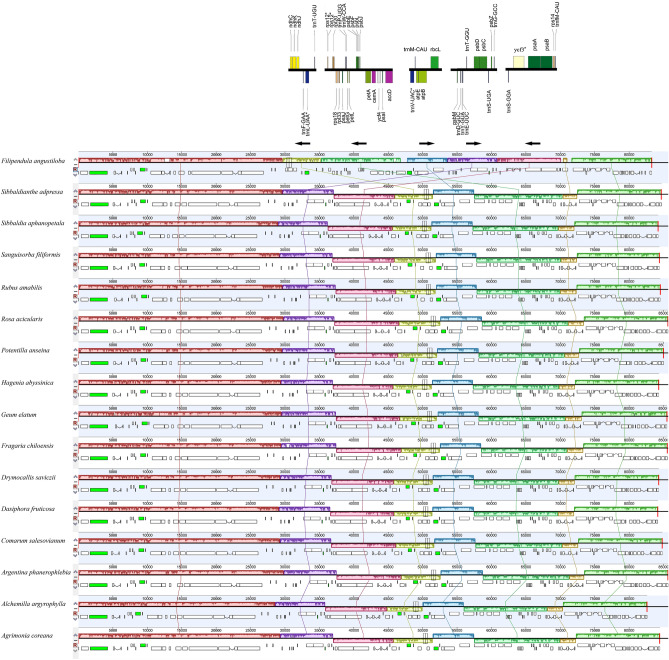
Structural variations between 15 representative genera of Rosoideae and *Filipendula* plastomes.

However, we found that *Filipendula* cp genomes exhibited the structural differences when compared with those of other genera of Rosoideae. At first, *Filipendula* cp genomes had a smaller gene number and three genes (*rps4, rpl2* and *rpl32*) were absent when compared to other genera of Rosoideae (Fig. 4). In addition, the gene order in three sequence blocks (*ndhC* and *trnT*-*UGU*, *rps12* and *accD*, *trnS*-*GGA* and *trnfM*-*CAU*) of other genera of Rosoideae plants were highly conserved, whereas those of *Filipendula* cp genomes significantly differed (Fig. 4). Further analysis indicated that a minimum of three inversions occurred within cp genomes of *Filipendula* species (Fig. 4). Besides, *Filipendula* species had a plesiomorphic gene order similar to other genera of Rosoideae plants in two blocks of *psbM* and *trnG*-*GCC*, *trnV*-*UAC* and *rbcL*. However, these two blocks had the obvious changes in location within the cp genomes of *Filipendula* when compared to those of other genera of Rosoideae plants (Fig. 4). Such transpositions of these blocks caused to the divergent chloroplast gene order between *Filipendula* plants and other genera of Rosoideae plants (Fig. 4). Therefore, the cp genomes of *Filipendula* species exhibited the considerable differences in structure from those of other genera of Rosoideae plants: a minimum of 3 inversions, transpositions of two blocks within the LSC and gene losses.

**Figure 5:**
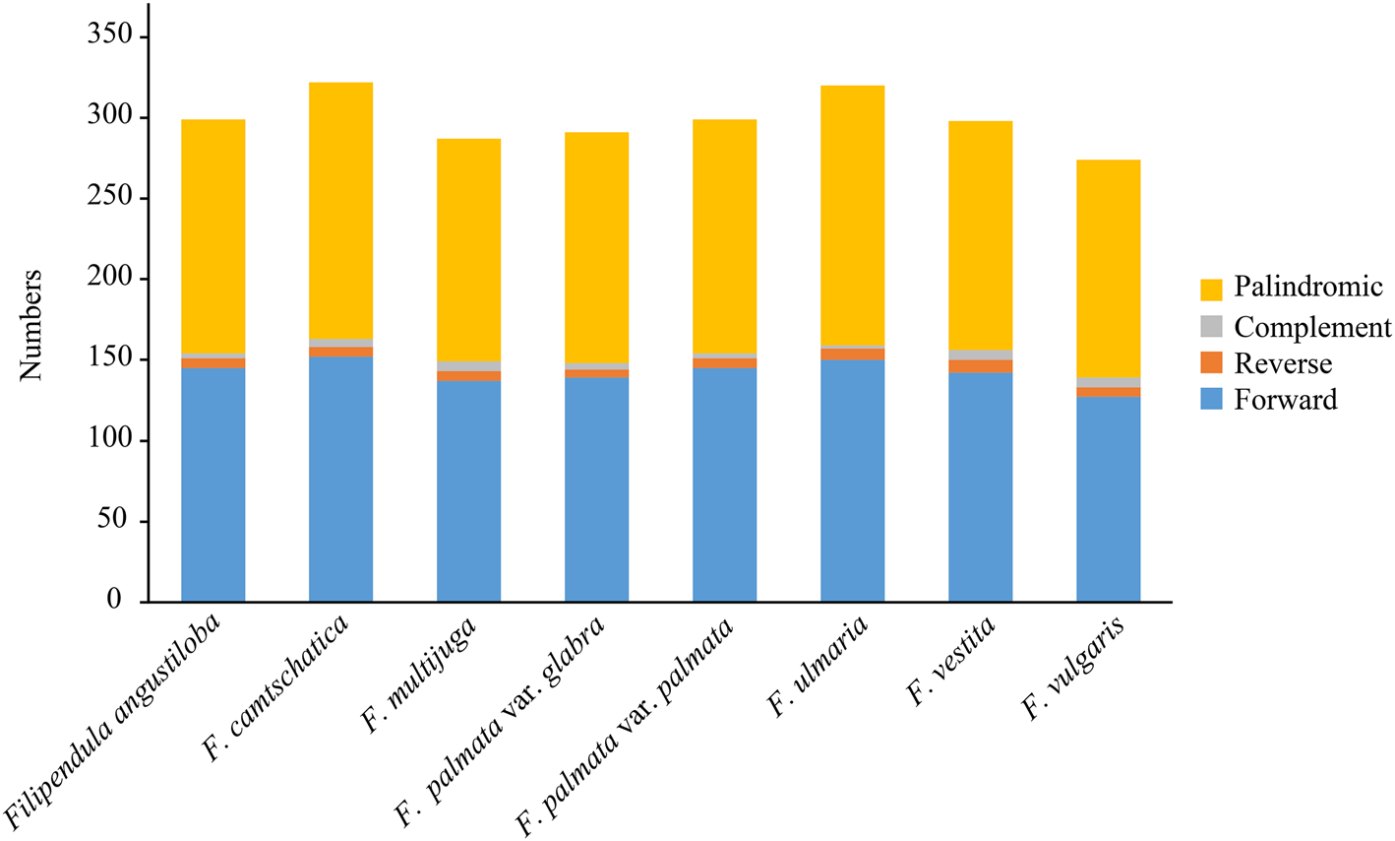
Number of four type repeats examined in eight *Filipendula* chloroplast genomes.

Repeats in plastome may be associated with the endpoints of inversion^5^. In present study, four types of repeats (palindromic repeats, forward repeats, reverse repeat and complement repeats) were detected in *Filipendula* cp genomes. The total number of repeats varied from 273 to 321 (Fig. 5), which outnumbered other species of Rosaceae (i.e. *Sorbus*)^26^. *Filipendula camtschatica* had the most abundant repeats, including 152, 6, 5 and 158 forward, reverse, complement and palindromic repeats, respectively (Fig. 4). Similarly, forward and palindromic repeats became two major repeat types in other six *Filipendula* species (Fig. 4). Although complementary repeats (2–6) and reverse repeats (5–8) had the small number, they were observed in each *Filipendula* species (Fig. 5). The majority of the repeats were found in intergenic regions (Table S2). Some repeats were found in coding or intron sequences of several genes, such as *trnG*-*UCC*, *trnG*-*GCC*, *trnL*-*UAA*, *accD*, *psaA*, *psaB*, *clpP*, *ycf1*, *ycf2*, *ycf3*, *ycf4*, *petB*, *ndhF* and *trnL*-*UAA* (Table S2). Interestingly, all the genes except *trnG*-*UCC*, *ycf1*, *ycf2*, *petB* and *ndhF* were located in three inversion and two transposition blocks (Fig. 5). Additionally, among six genes of reversion endpoints, only *accD* contained the repeats, none of repeats were observed in the remainder (*ndhC, rps12, trnfM-CAU, trnS-GGA* and *trnT-UGU*) (Table S2). It was worth mentioning that *rps12* was duplicated in the endpoint of *rps12-accD* inversion in *Filipendula* (Fig. 5).

### Genomic sequence divergence analysis in *Filipendula*

To better understand the sequence divergence of *Filipendula* species, eight whole plastomes were compared and used to analyse sequence identity with mVISTA program using the cp genome of *F. angustiloba* as a reference. The results indicated that the whole cp genomes of *Filipendula* species were relatively conserved, in which the LSC region exhibited the highest divergence, whereas the IR regions were the most conserved (Figure S2). In addition, the high sequence divergence mainly occurred in noncoding regions, whereas only several genes (i.e., *accD, clpP, ycf1* and *ycf2*) were found to be divergent in their coding regions (Figure S2).

**Figure 6:**
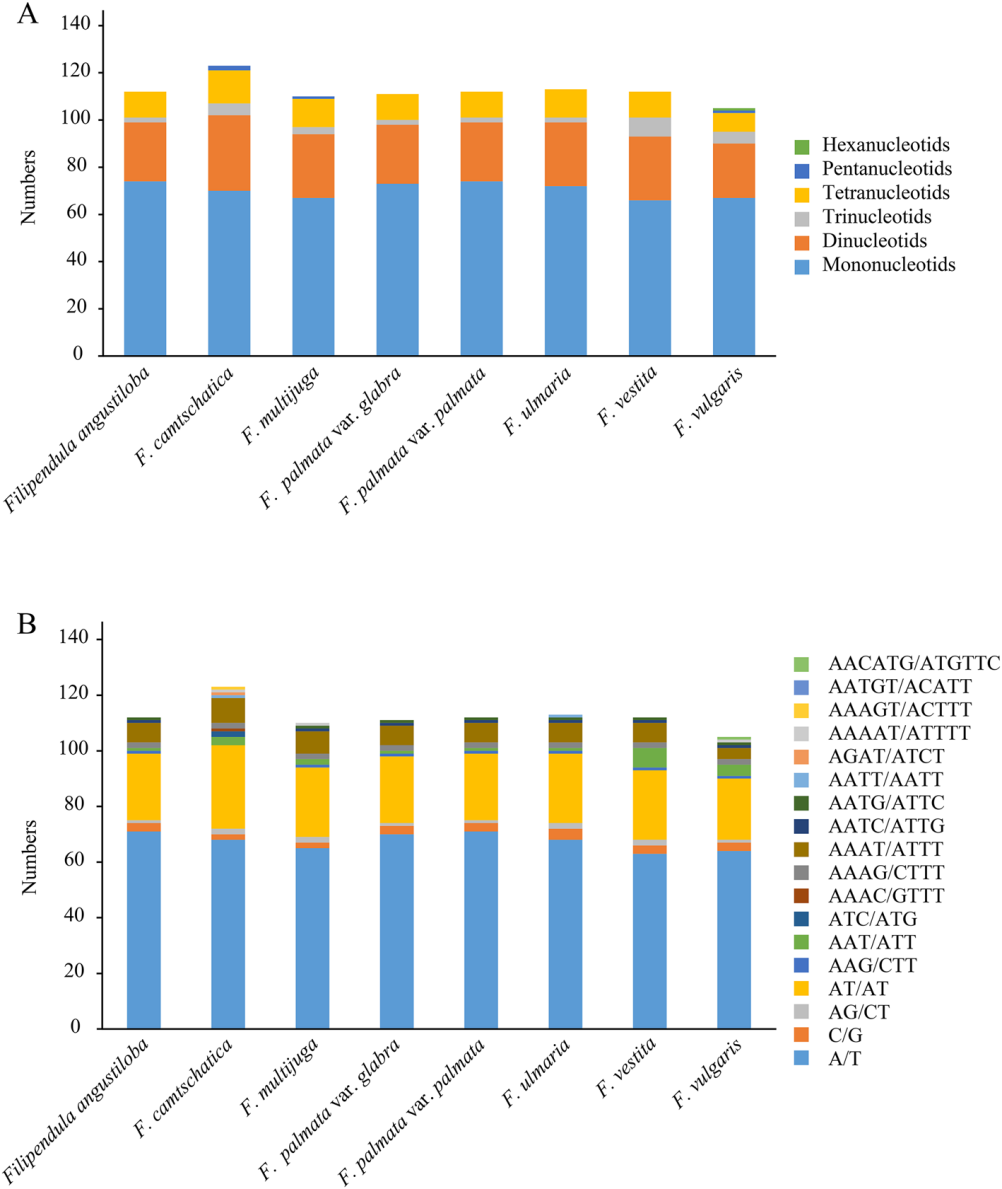
Frequency of six SSR types (**A**) and distribution of SSR sequences (**B**) examined in eight *Filipendula* chloroplast genomes.

SSRs are a class of short tandem repeats (1–6 bp) and highly polymorphic markers, which are widely distributed in the plastomes in plants and commonly used for species identification and phylogenetic analyses^27–29^. In this study, the mono-, di-, tri-, tetra-, penta- and hexa-nucleotide repeat units were analysed. *Filipendula* cp genomes were found to contain 105 (*F. vulgaris*) to 123 (*F. camtschatica*) SSRs (Fig. 6A, Table S3). Most of the SSRs were mononucleotide repeats (66.07%, 56.91%, 60.91%, 65.77%, 66.07%, 63.72%, 58.93% and 63.81% in* F. angustiloba*, *F. camtschatica*, *F. multijuga*, *F. palmata* var. *glabra*, *F. palmata* var. *palmata*, *F. ulmaria*, *F. vestita* and *F. vulgaris*, respectively), which mainly made up of A and T nucleotides (Fig. 6A,B). Dinucleotide repeats were the second abundant SSRs with the major constitution of AT/AT nucleotides (Fig. 6A,B). Trinucleotids and tetranucleotide repeats were small in number, but both repeats were observed in each *Filipendula* plastomes (Fig. 6A,B, Table S3). By contrast, pentanucleotids and hexanucleotids were found in only few *Filipendula* plastomes. For example, few pentanucleotide repeats were only found in *F. multijuga*, *F. camtschatica* and *F. vulgaris* and one hexanucleotide repeat was only found in *F. vulgaris* (Fig. 6A).

**Figure 7:**
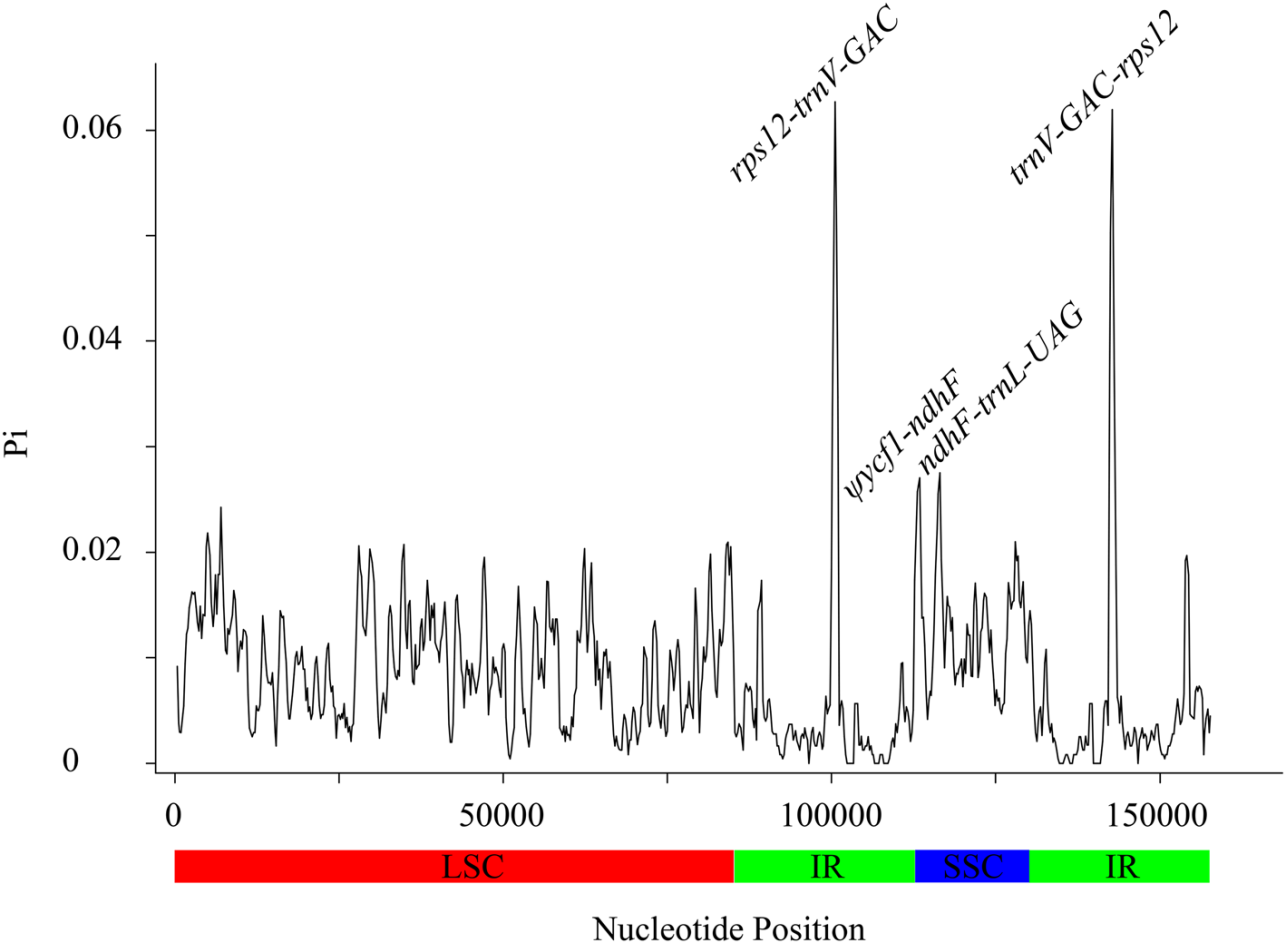
Sliding window analysis of Pi values among cp genomes of seven *Filipendula* species. X-axis, position of the midpoint of a window; Y-axis, nucleotide diversity of each window. (Window length: 600 bp, step size: 200 bp).

Besides, sliding window analysis was conducted to reveal the highly variable regions in eight *Filipendula* cp genomes. The average value of nucleotide diversity (Pi) over the entire cp genome was 0.005, indicating the whole cp genome was relatively conserved (Fig. 7). This result was consistent with the mVISTA result (Figure S2). In addition, we found that the high variability mainly occurred in noncoding regions. Four mutational hotspots with pi values greater than 0.02 were identified, namely *ψycf1-ndhF, rps12-trnV-GAC, ndhF-trnL-UAG,* and *trnV-GAC-rps12* (Fig. 7). Of four variable regions, *rps12-trnV-GAC* and *trnV-GAC-rps12* from two IR regions had the highest pi value. Based on these results, the noncoding regions exhibited the higher variability and divergence than the protein-coding regions. And then, selective signatures were determined by the ratios of non-synonymous (Ka) to synonymous (Ks) substitution rates on the 76 unique protein sequences. Our results demonstrated that the ratios of Ka/Ks of the majority of genes in these *Filipendula* species were less than 1, suggesting these PCGs were under strong purifying selection (Table S4). Two genes (*matK* and *rps8*) with Ka/Ks ratios more than 1 were under positive selection (Table S4).

### Phylogenetic and molecular dating analysis of *Filipendula*

The structural rearrangement of chloroplast genome is usually used for reconstructing phylogenies of plants^5^. Based on our results, the overall structure of cp genome was highly conserved in seven *Filipendula* species (including two varieties). Under this case, the high homoplasy of cp genome structure was not used for phylogenetic analysis. Nevertheless, the cp genome of genus *Filipendula* generated gene loss, transposition and inversion, whereas the other genera of Rosoideae were lack of these structural changes. Two previous studies have given an identical support for *Filipendula* as the first clade to split off the rest of Rosoideae in the nuclear and plastome trees^21,30^. Therefore, *Filipendula* was the basal clade in the Rosoideae probably because the gene loss, transposition and inversion mark an ancient evolutionary split in this subfamily.

**Figure 8:**
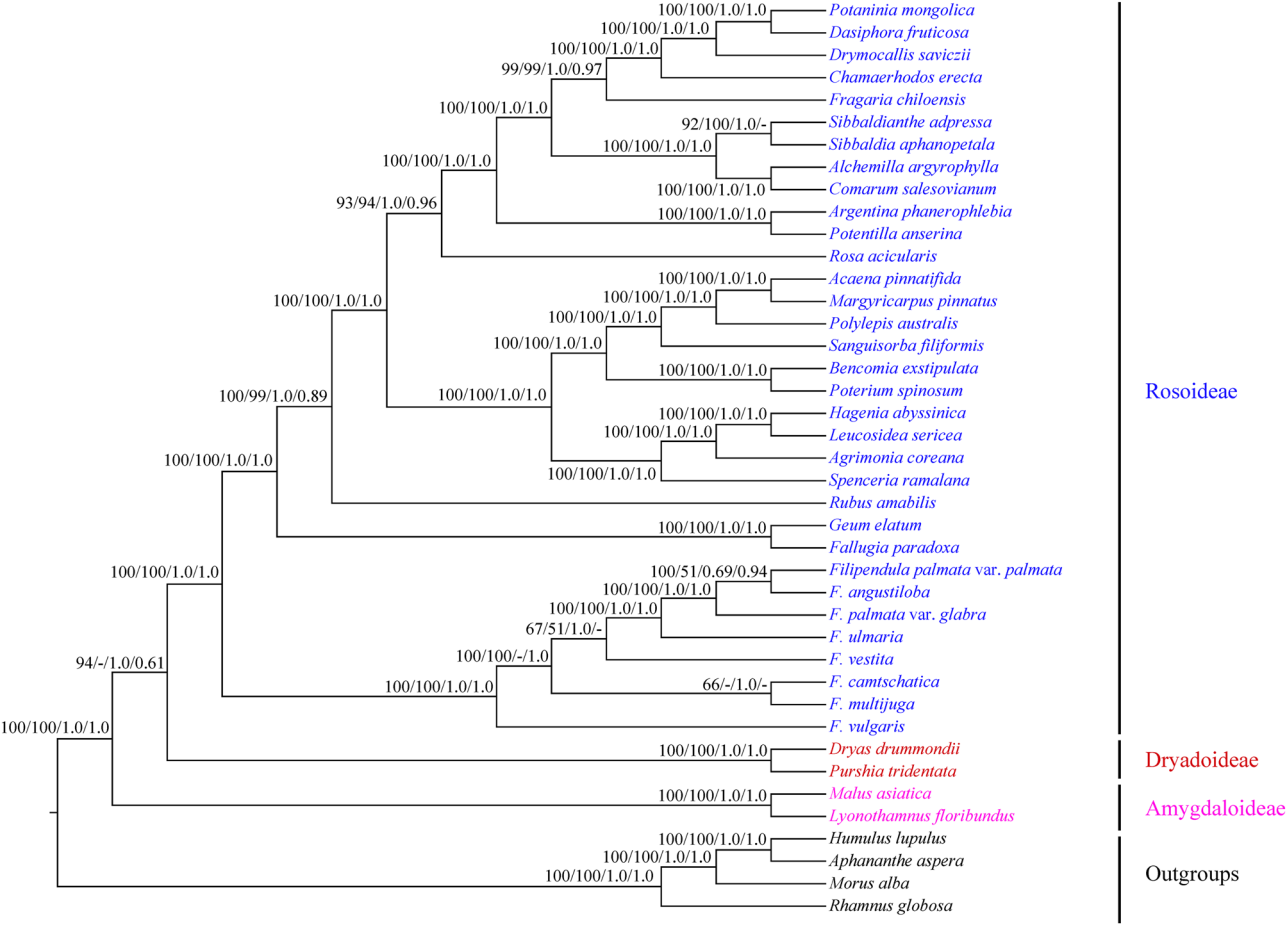
The phylogenetic tree of seven *Filipendula* species and 29 species of three subfamilies of Rosaceae based on PCG data. Numbers at nodes correspond to the support values of ML, MP, BI and ASTRAL, respectively.

Besides, sequence divergence generated a large number of genetic variations in eight *Filipendula* cp genomes, which can be used for reconstructing the phylogeny of *Filipendula*. In this study, all PCG sequences were used to infer the phylogenetic relationships within this genus by ML, MP, BI and ASTRAL methods. The results indicated that these trees formed the major identical topology (Fig. 8). The phylogenetic analyses revealed that *Filipendula* was the basal genus in Rosoideae, which was consistent with previous results^20,21^. Infrageneric relationship of *Filipendula* had been resolved two major clades (Fig. 8). One clade contained *F. vulgaris* (the type species) with high support values (i.e. 100% BS and 1.0 PP). The remaining species clustered into two sister clades (Fig. 8). One sister group contained *F. camtschatica* and *F. multijuga* and other five *Filipendula* species or varieties formed another group (Fig. 8). In the previous study, phylogenetic analyses also resolved two major lineages within *Filipendula* based on one nucleotide segment (ITS)^10^. However, *F. occidentalis* from North America was the basal species and the others from Asia and Europe clustered into two sister clades^10^. Therefore, we will greatly expand the sampling to better understand the phylogenetic relationships within *Filipendula*.

**Figure 9:**
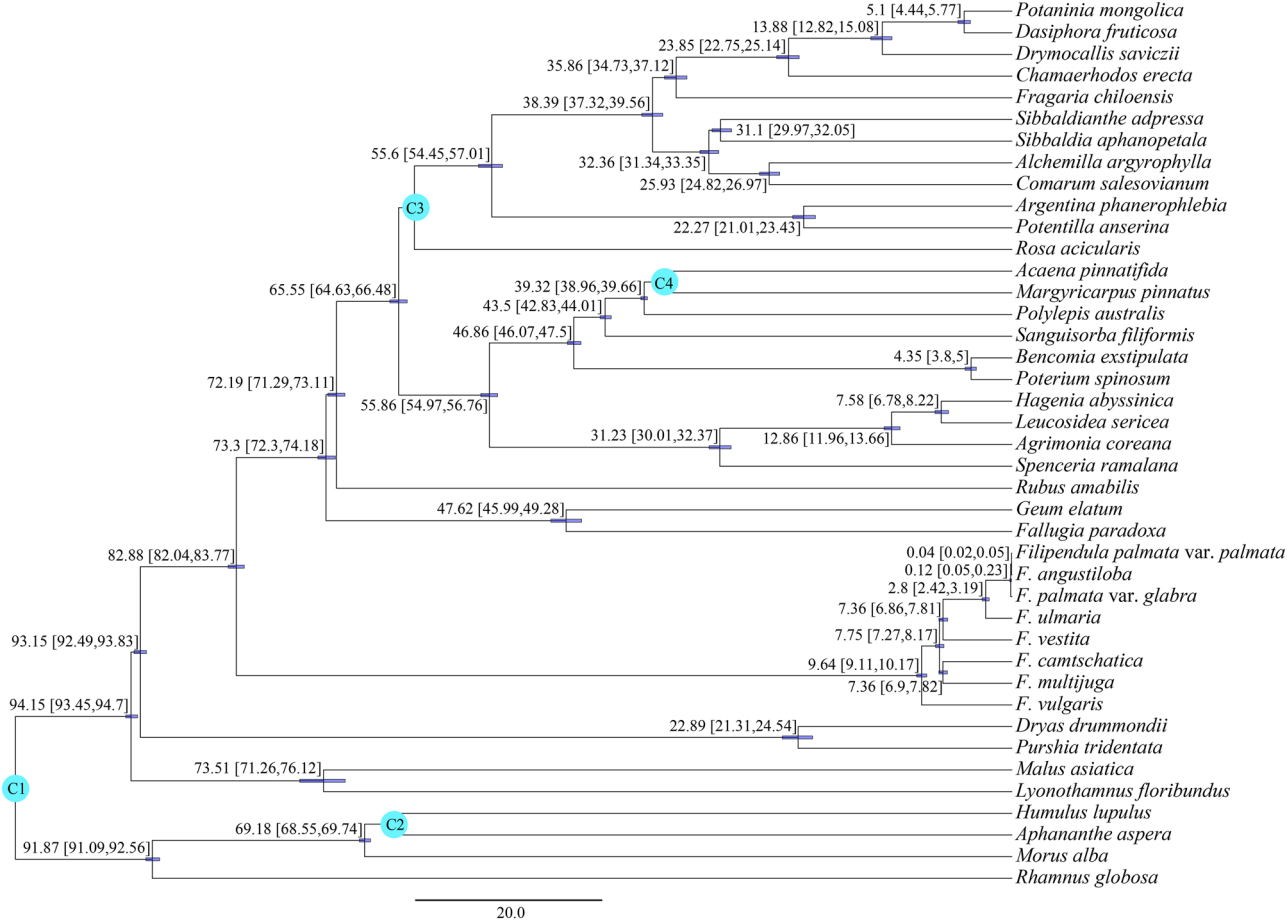
Divergence times estimation of 40 species of Rosaceae based on PCG data. The divergence times are shown near each node. Blue bars represent 95% high posterior density for the estimated mean dates. C1-C4 in the blue green circle represent calibration points.

The divergence time between *Filipendula* and other genera of Rosoideae was estimated at 82.88 Ma (82.04–83.77 Ma, 95% HPD) in the Cretaceous (Fig. 9). After that, *Filipendula* located on a long branch, implying this genus had an evolutionary history different from other genera of Rosoideae. As shown in Fig. 9, the age of the most recent common ancestor of *Filipendula* was estimated at about 9.64 Ma (9.11–10.17 Ma, 95% HPD) in the late Miocene. In contrast, intergeneric diversity times of other genera of Rosoideae occurred from 73.3 Ma to 4.35 Ma. Previous studies have demonstrated that the diversification of Rosaceae increased at two different periods. The rapid initial diversity occurred in the late Cretaceous and the second shift occurred in the early Oligocene onwards^21^. Based on our results, genus *Filipendula* had an early origin but late diversification during evolutionary process, which was apparently different from other genera of Rosoideae.

## Discussion

The chloroplast genome has been used as a powerful marker for investigating plant evolution and phylogenetic analysis^5^. Several cases have been published comparing genomes of taxa among which the structural changes occurred in cp genomes of the basal members at the different taxonomic levels^2,5^. In the present study, the cp genomes of several *Filipendula* species in the basal clade of subfamily Rosoideae were first analysed. Our results indicated that eight *Filipendula* cp genomes share several common features with those of other genera of Rosoideae. For example, they have a typical quadripartite structure of cp genomes and similar GC content with most land plants^26^. The cp genome organization was highly conserved in the tested *Filipendula* species, but these cp genomes exhibited the apparent changes in gene order and orientation when compared with those of other genera of Rosoideae. Our results revealed that gene loss, inversion and transposition contributed to the structural changes between *Filipendula* species and the remainder of Rosoideae. Therefore, the phenomenon of structural changes (rearrangements) occurred in *Filipendula* species of the basal linage of Rosoideae.

The expansion and contraction or loss of IR can disrupt gene order, whereas the stability of IR may facilitate the gene order conservation^5^. In this study, the nearly unchanged boundaries of IR/SC contributed to reduce gene order changes of *Filipendula* cp genomes. In addition, gene/intron loss is considered as one of three classes of gene order changes in the land plant cp genomes^5^. In the present study, the gene loss events might occur in the *Filipendula* cp genomes because several genes were not found in their cp genomes. However, one gene absence (*rpl14*) within *Filipendula* was restricted to individual species (*F. camtschatica*). Therefore, we inferred that gene loss events might have continued to occur during *Filipendula* plant diversification. Inversion is the most common mechanism leading to gene order changes^5^. In this study, three inversions and two transpositions mainly contributed to the changes of order and orientation of 39 genes, which were involved in almost all functional classifications. Most chloroplast genes are often under the control of an operon; therefore, transcriptional regulation of these genes might be affected by the changes of gene order and orientation. Usually, both endpoints of inversion occur in non-coding regions, in which no genes are disrupted. In the study, one inversion occurred with the endpoint in *accD*. This gene not only contained the sequence repeats, but also exhibited the high sequence polymorphism. Therefore, we inferred that *accD* might be active in contribute to genomic rearrangement and sequence divergence of *Filipendula* plants.

The structural rearrangements have led to the low levels of homoplasy of cp genomes between *Filipendula* and the remainder of Rosoideae. In this study, the sequences of all unique PCGs were used to construct the phylogenetic tree. Our results indicated that *Filipendula* was indeed located in the basal linage of Rosoideae, which was consistent with the previous results^20–22^. Besides, we found that the gene order and orientation were conserved between the other genera of Rosoideae and the representative species of Dryadoideae, Amygdaloideae. Therefore, we inferred that the structural rearrangement of cp genomes should be an independent evolutionary event within *Filipendula* after divergence from the other genera of Rosoideae. Meanwhile, *Filipendula* species showed the different diversity periods from other genera of Rosoideae, implying that *Filipendula* species might experience the different evolutionary process. In the present study, infrageneric relationship of *Filipendula* was highly supported using PCGs, which was a little different from the segment tree^10^. In previous study, more samples were used to construct the phylogenetic tree, including the species from North America, Asia and Europe^10^. Therefore, the comprehensive phylogenetic relationship might be better understood by sampling more species within *Filipendula*.

## Materials and methods

### DNA extraction, sequencing, assembly and annotation

A total of seven *Filipendula* species were used, including *F. vestita, F. ulmaria, F. palmata* (including two varieties, *F. palmata* var. *palma* and *F. palmata* var. *glabra*), *F. angustiloba, F. vulgaris*, *F. camtschatica* and *F. multijuga* in this study. Of them, the former five species were collected from the different provinces of China and the last two from UK and Japan, respectively. The raw sequencing reads of *F. vulgaris* was downloaded from the NCBI SRA database. The detailed information was shown in Table S5. All the voucher specimens we collected were deposited in Herbarium, Kunming Institute of Botany, CAS (KUN) or Royal Botanic Garden Edinburgh Herbarium (E). The silica gel-dried leave of each species was used to extract the genomic DNA by the modified CTAB method and the constructed libraries were sequenced by Illumina NovaSeq PE150 platform.

The high-quality reads of the cp genome data were de novo assembled into the contigs using SPAdes software^31^, which were further circulated using Bandage software^32^. The genome annotation was implemented by GeSeq software^33^ using *Potentilla* spp. as references with 65% and 80% similarity to proteins and tRNA (or rRNA) genes, respectively. The annotated and circular plastome was drawn using OGDRAW program^34^. Besides, the raw data of cp genome of *F. vulgaris* (type species) were downloaded (SRA: ERR5554718) and analyzed using the same method as described in six species. All methods of experimental research on plants were performed in accordance with the relevant institutional, national, and international guidelines and legislation.

### Comparative analysis of chloroplast genomes

Four different repeat types, including forward, palindrome, reverse and complement sequences were analyzed and six microsatellites (simple sequence repeats, SSRs), including mono-, di-, tri-, tetra-, penta-, and hexa-nucleotide repeat units were identified in seven *Filipendula* species as previously described^26^. After the plastid genome sequences of seven *Filipendula* species were aligned using MAFFT v.6.833^35^ using the default settings, multiple alignments were used to analyze infrageneric genome collinearity using the Mauve software^36^. In this study, the representative species from 15 genera of Rosoideae, including *Agrimonia coreana, Alchemilla argyrophylla, Argentina phanerophlebia, Comarum salesovianum, Dasiphora fruticose, Drymocallis saviczii, Filipendula angustiloba, Fragraria chiloensis, Geum elatum, Hagenia abyssinica, Potentilla anserina, Rosa acicularis, Rubus amaabilis, Sanguisorba filiformis, Sibbaldia aphanopetala and Sibbaldianthe adpressa* were used for intergeneric collinearity analyses. Moreover, the comparative review of the whole genome alignment of seven *Filipendula* species was visualized using the mVISTA program^37^ using *F. angustiloba* as reference sequence. The online IRscope tool^38^ was used to analyze the joint site information of cp genome.

The sliding window analysis of nucleotide diversity (Pi) of cp genome was performed by DNASP 5.0 program^39^. The window length and step size were set to 600 and 200 bp, respectively. The ratio of the nonsynonymous (Ka) and synonymous (Ks) substitution rates was used to calculate the selective pressure between orthologous genes of cp genome of *Filipendula* species. The coding gene sequences were selected from each cp genome and then aligned by using by MAFFT v.6.833^35^. The resulted alignment was used to calculate the ratio of Ka/Ks using KaKs_Calculator 3.0^40^.

### Phylogenetic analysis and divergence time estimates

A total of seven *Filipendula* species (including two varieties of *F. palmata*) were used to construct the phylogenetic tree. Four species from other families of Rosales (two species from Cannabaceae, and one species from Moraceae and Rhamnaceae, respectively) and 29 species of three subfamilies of Rosaceae (one representative species of 25 other genera of Rosoideae, 2 species from Dryadoideae and 2 species from Amygdaloideae) were used as outgroups (Table S6). In this study, 76 protein-coding gene sequences were extracted on the basis of their annotation. All these sequences were aligned using MAFFT v.6.833^35^. We constructed the phylogenies using the concatenated and coalescent methods. For the concatenated analysis, all aligned PCG sequences were concatenated to a single alignment dataset for phylogenetic inference using maximum likelihood analysis (ML), Bayesian inference (BI), maximum parsimony (MP) methods. Briefly, the maximum likelihood (ML) analysis was performed using RAxML v7.2.6^41^ under GTRGAMMA model for 1000 bootstrap^42^. Bayesian inference (BI) was performed using MrBayes v3.1.2^43^. The Markov Chain Monte Carlo (MCMC) analysis was run for 2 million generations. The trees were sampled at every 100 generations and the first 25% trees were discarded as burn-in. Finally, the majority-rule consensus tree was generated by the remaining trees with posterior probability (PP) values for each node. Maximum parsimony (MP) analysis was run in PAUP (v4.0b10)^44^, using heuristic search with 1000 bootstrap replicates and tree bisection-reconnection (TBR) branch swapping. For the coalescent analysis, each PCG was used to construct a ML tree as above described. All generated gene trees were used to estimate the species tree with ASTRAL^45^ in Phylosuit^46^.

From the best ML tree, we generated 1000 bootstrap replicates to produce a dated phylogeny with a 95% confidence interval (CI) on the age at the nodes using TREEPL^47^, following the guide by Maurin^48^. We considered 90 and 106.5 Ma as the minimum- and maximum-age calibrations for the stem of Rosaceae as suggested by Zhang et al.^21^. Three fossil calibrations were also used as minimum-age calibrations assigned to internal nodes (all outside our study clades) (Table S7).

## Conclusions

The complete chloroplast genomes of seven *Filipendula* species were analysed in this study. The genome structure and gene content within *Filipendula* were rather conserved. However, gene loss, transpostion and inversion were observed in the cp genomes of *Filipendula* when compared with those of other genera of Rosoideae. Sequence divergence mainly occurred in noncoding regions, in which numbers of SSRs and four mutational hotspots were identified in each *Filipendula* species. The phylogenetic and molecular dating analyses showed that *Filipendula* was divergent from other genera of Rosoideae about 82.88 Ma (82.04–83.77 Ma, 95%HPD). And seven *Filipendula* species were split at 9.64 Ma (9.11–10.17 Ma, 95%HPD) into two major clades. The results provided the basis for the study of the evolutionary history and phylogenetic analysis of *Filipendula*.

### Supplementary Information

Supplementary Information.

## Data Availability

The datasets generated and analyzed during the current study can be accessed in the NCBI GenBank database, and the accession numbers of seven *Filipendula* species are listed in Table S6.
