# Supplementary material for: Comparative and phylogenetic analyses of the chloroplast genomes of Filipendula species (Rosoideae, Rosaceae)
Source: Sci Rep. 2023 Oct 18;13:17748. doi: 10.1038/s41598-023-45040-3 (PMC10584953; doi:10.1038/s41598-023-45040-3)
Supplement: Supplementary file 1 — Supplementary Information. [file 41598_2023_45040_MOESM1_ESM.zip › supplementary files/Table S1 Functional classification of chloroplast genome genes in Filipendula.docx]

**Table S1**. Functional classification of chloroplast genome genes in *Filipendula*

| **Classification** | **Gene group** | **Gene name** |
| --- | --- | --- |
| Gene related to photosynthesis | Photosystem Ⅰ | *psaA, psaB, psaC, psaI, psaJ* |
|  | Photosystem Ⅱ | *psbA, psbB, psbC, psbD, psbE, psbF, psbH, psbI, psbJ, psbK, psbL, psbM, psbN, psbT, psbZ* |
|  | Cytochrome b/f complex | *petA, petB*^a^*, petD*^a^*, petG, petL, petN* |
|  | ATP synthase | *atpA, atpB, atpE, atpF*^a^*, atpH, atpI* |
|  | NADH-dehydrogenase | *ndhA*^a^*, ndhB**^a^*, ndhC, ndhD, ndhE, ndhF, ndhG, ndhH, ndhI, ndhJ,*  *ndhK* |
|  | Subunit of rubisco | *rbcL* |
| Self replication gene | RNA polymerase | *rpoA, rpoB, rpoC1*^a^*, rpoC2* |
|  | Small subunit of ribosome | *rps2, rps3, rps7*, rps8, rps11, rps12**^b^*, rps14, rps15, rps16*^a^*, rps18, rps19* |
|  | Large subunit of ribosome | *rpl14, rpl16*^a^*, rpl20, rpl22, rpl23*, rpl33, rpl36* |
|  | tRNA | *trnA-UGC**^a^*, trnC-GCA, trnD-GUC, trnE-UUC, trnF-GAA,*  *trnfM-CAU, trnG-GCC, trnG-UCC*^a^*, trnH-GUG, trnI-CAU*, trnI-GAU**^a^*, trnK-UUU*^a^*, trnL-CAA*, trnL-UAA*^a^*, trnL-UAG, trnM-CAU, trnN-GUU*, trnP-UGG, trnQ-UUG, trnR-ACG*, trnR-UCU, trnS-GCU, trnS-GGA, trnS-UGA, trnT-GGU, trnT-UGU,*  *trnV-GAC*, trnV-UAC*^a^*, trnW-CCA, trnY-GUA* |
|  | rRNA | *rrn4.5*, rrn5*, rrn16*, rrn23** |
| Other gene | Mature enzyme gene | *matK* |
|  | Translational initiation  factor | *infA* |
|  | C-type cytochrome synthesis gene | *ccsA* |
|  | Subunits of Acetyl-CoA-carboxylase | *accD* |
|  | Envelop membrane  protein | *cemA* |
|  | Protease | *clpP* |
| Unknown gene | Conserved open reading frames | *ycf1, ycf2*, ycf3*^b^*, ycf4* |

Note: *indicates duplicated gene; ^a^Gene contains a single intron; ^b^Gene contains two introns.
