# Supplementary material for: Comparative and phylogenetic analyses of the chloroplast genomes of Filipendula species (Rosoideae, Rosaceae)
Source: Sci Rep. 2023 Oct 18;13:17748. doi: 10.1038/s41598-023-45040-3 (PMC10584953; doi:10.1038/s41598-023-45040-3)
Supplement: Supplementary file 1 — Supplementary Information. [file 41598_2023_45040_MOESM1_ESM.zip › supplementary files/Table S5 The information of seven Filipendula species used in this study.docx]

**Table S5**. The information of seven *Filipendula* species or varieties used in this study.

| **Species** | **Geographical origin** | **Voucher number** |
| --- | --- | --- |
| *F. angustiloba* | Jilin Province, China | AnHC0533 |
| *F. camtschatica* | Royal Botanic Garden Edinburgh, UK (cultivated) | RBGE 20022321 A |
| *F. multijuga* | Japan | Yi12645 |
| *F. palmata* var. *glabra* | Nei Mongol Autonomous Region, China | Huangxw2254 |
| *F. palmate* var. *palmata* | Heilongjiang province, China | YNYS1103 |
| *F. ulmaria* | Xinjiang Uygur Autonomous Region, China | 16CS13860 |
| *F. vestita* | Yunnan province, China | Yi15399 |
|  |  |  |
