# Supplementary material for: Comparative and phylogenetic analyses of the chloroplast genomes of Filipendula species (Rosoideae, Rosaceae)
Source: Sci Rep. 2023 Oct 18;13:17748. doi: 10.1038/s41598-023-45040-3 (PMC10584953; doi:10.1038/s41598-023-45040-3)
Supplement: Supplementary file 1 — Supplementary Information. [file 41598_2023_45040_MOESM1_ESM.zip › supplementary files/Table S6 The information of species used for reconstruction of phylogenetic tree.docx]

**Table S6.** The information of species used for reconstruction of phylogenetic tree.

| **Family** | **Subfamily** | **Taxon** | **Accession number** |
| --- | --- | --- | --- |
| Cannabaceae | | *Aphananthe aspera* | MH118117 |
| Cannabaceae | | *Humulus lupulus* | KT266264 |
| Moraceae |  | *Morus alba* | MW465954 |
| Rhamnaceae | | *Rhamnus globosa* | MT360052 |
| Rosaceae | Amygdaloideae | *Lyonothamnus floribundus* | KY420005 |
| Rosaceae |  | *Malus asiatica* | MW115593 |
| Rosaceae | Dryadoideae | *Dryas drummondii* | KY419952 |
| Rosaceae |  | *Purshia tridentata* | KY420000 |
| Rosaceae | Rosoideae | *Acaena pinnatifida* | KY419984 |
| Rosaceae |  | *Agrimonia coreana* | MW659450 |
| Rosaceae |  | *Alchemilla argyrophylla* | MT382661 |
| Rosaceae |  | *Argentina phanerophlebia* | MT114192 |
| Rosaceae |  | *Bencomia exstipulata* | MG682353 |
| Rosaceae |  | *Chamaerhodos erecta* | KY420001 |
| Rosaceae |  | *Comarum salesovianum* | MT017928 |
| Rosaceae |  | *Dasiphora fruticosa* | MF683841 |
| Rosaceae |  | *Drymocallis saviczii* | MT178809 |
| Rosaceae |  | *Fallugia paradoxa* | KY419999 |
| Rosaceae |  | ***Filipendula angustiloba*** | OP963798 |
| Rosaceae |  | ***F. camtschatica*** | OQ396769 |
| Rosaceae |  | ***F. multijuga*** | OP963792 |
| Rosaceae |  | ***F. palmata* var. *glabra*** | OP963796 |
| Rosaceae |  | ***F. palmata* var. *palmata*** | OP963793 |
| Rosaceae |  | ***F. ulmaria*** | OP963797 |
| Rosaceae |  | ***F. vestita*** | OP963794 |
| Rosaceae |  | *F. vulgaris* | ERR5554718 |
| Rosaceae |  | *Fragaria chiloensis* | MW537844 |
| Rosaceae |  | *Geum elatum* | MT982432 |
| Rosaceae |  | *Hagenia abyssinica* | KX008604 |
| Rosaceae |  | *Leucosidea sericea* | KY419929 |
| Rosaceae |  | *Margyricarpus pinnatus* | KY419972 |
| Rosaceae |  | *Polylepis australis* | KY419989 |
| Rosaceae |  | *Potaninia mongolica* | MN691039 |
| Rosaceae |  | *Potentilla anserina* | OW176989 |
| Rosaceae |  | *Poterium spinosum* | KY419948 |
| Rosaceae |  | *Rosa acicularis* | MK714016 |
| Rosaceae |  | *Rubus amabilis* | MN652918 |
| Rosaceae |  | *Sanguisorba filiformis* | MF678800 |
| Rosaceae |  | *Sibbaldia aphanopetala* | MT178810 |
| Rosaceae |  | *Sibbaldianthe adpressa* | MT114191 |
| Rosaceae |  | *Spenceria ramalana* | KY419995 |

Note: The species or varieties with bold were newly sequenced in this study.
