# Supplementary material for: Comparative and phylogenetic analyses of the chloroplast genomes of Filipendula species (Rosoideae, Rosaceae)
Source: Sci Rep. 2023 Oct 18;13:17748. doi: 10.1038/s41598-023-45040-3 (PMC10584953; doi:10.1038/s41598-023-45040-3)
Supplement: Supplementary file 1 — Supplementary Information. [file 41598_2023_45040_MOESM1_ESM.zip › supplementary files/Table S7 Macrofossils of Rosaceae used as calibration points for molecular dating.docx]

**Table S7.** Macrofossils of Rosaceae used as calibration points for molecular dating.

| **Node** | **Anchor fossil** | **Assigned date (Ma)** | **Reference(s)** |
| --- | --- | --- | --- |
| C1 | Rosales crown | 106.5–90 | Crepet & Nixon (1996), Wang et al. (2009), Li et al. (2015) |
| C2 | *Aphananthe cretacea* | 66.0 | Knobloch & Mai (1986) |
| C3 | *Rosa* *germerensis* | 47.8 | Edelman (1975) |
| C4 | *Acaena* sp. | 37.2 | Zetter et al. (1999) |
