# Supplementary figures and images for: Comparative and phylogenetic analyses of the chloroplast genomes of Filipendula species (Rosoideae, Rosaceae)
Source: Sci Rep. 2023 Oct 18;13:17748. doi: 10.1038/s41598-023-45040-3 (PMC10584953; doi:10.1038/s41598-023-45040-3)

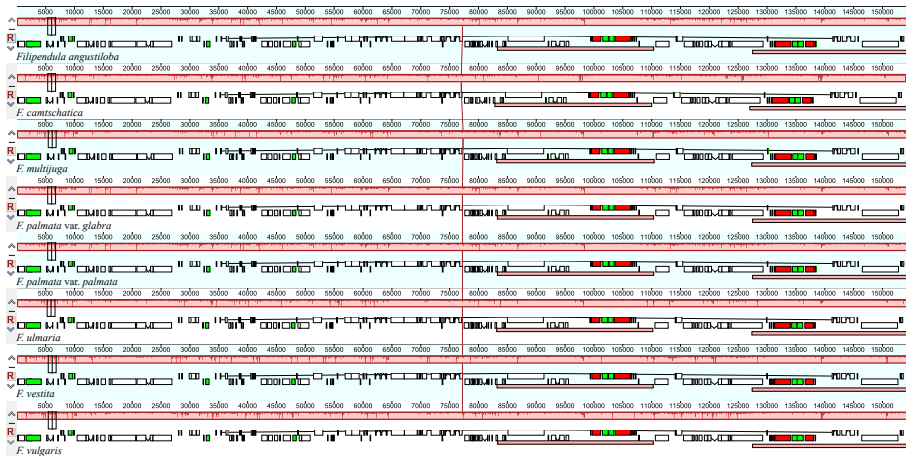

Figure S1 Collinearity analysis of chloroplast genomes across seven *Filipendula* species.

Supplement: Supplementary file 1 — Supplementary Information. [file 41598_2023_45040_MOESM1_ESM.zip › supplementary files/Figure S1 .pdf]
