# Supplementary material for: Comparative and phylogenetic analyses of the chloroplast genomes of Filipendula species (Rosoideae, Rosaceae)
Source: Sci Rep. 2023 Oct 18;13:17748. doi: 10.1038/s41598-023-45040-3 (PMC10584953; doi:10.1038/s41598-023-45040-3)
Supplement: Supplementary file 1 — Supplementary Information. [file Figure S2.pdf › supplementary files/Figure S2.pdf]

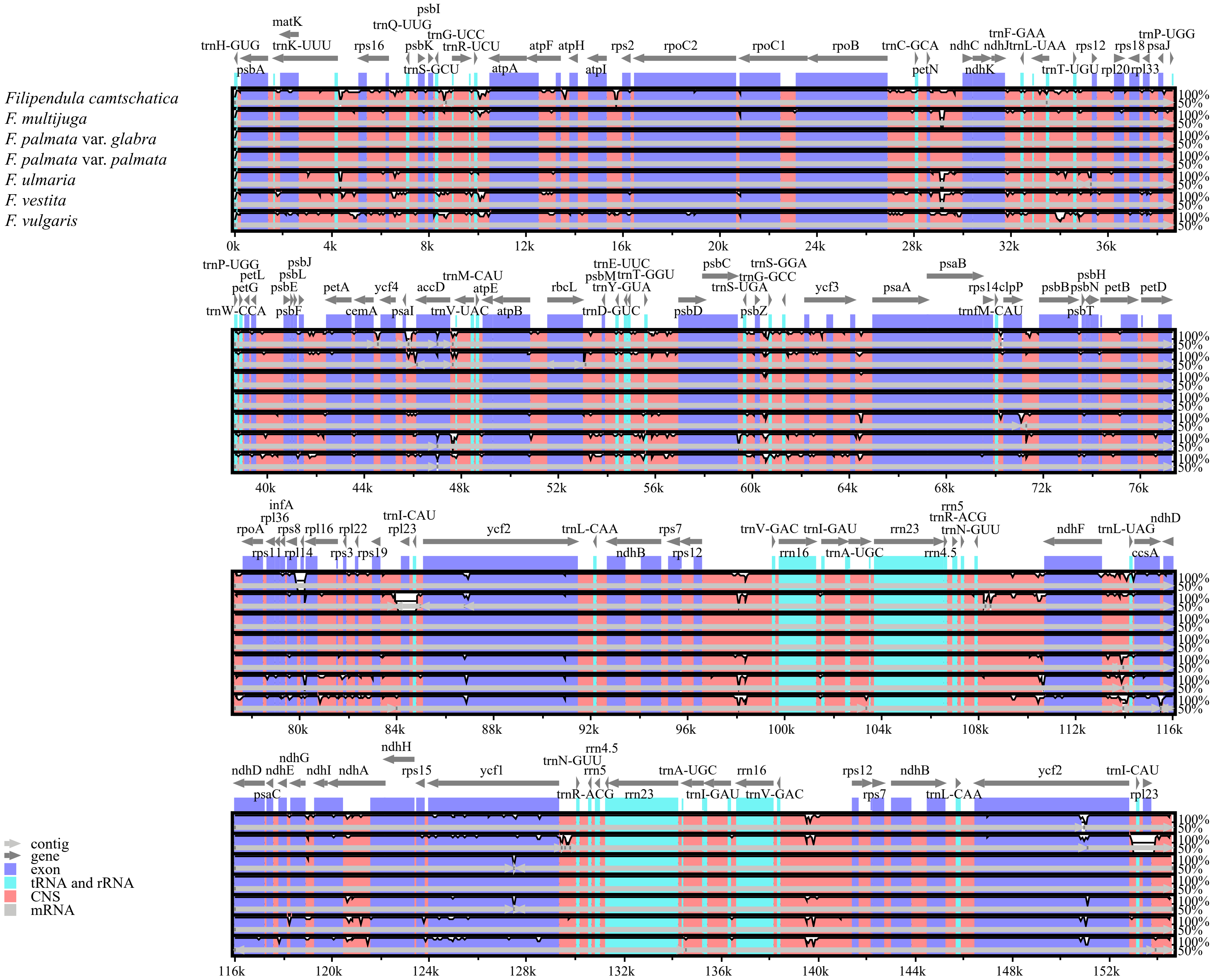

Figure S2 Sequence divergence of chloroplast genome sequences in seven *Filipendula* species.
